# Hydrogeological evaluation and sorption process of contaminants onto clay and sand as multi-barrier of a radioactive disposal site

**DOI:** 10.1038/s41598-026-62076-3

**Published:** 2026-07-20

**Authors:** Mahmoud M. Gouda, Ahmed A. Zaki

**Affiliations:** Radioactive Waste Management Department, Hot Laboratory and waste management center, Egyptian Atomic Energy Authority, P.O. Box 13759, Cairo, Egypt

**Keywords:** Saturated hydraulic conductivity, Barrier layer, Acidic conditions, Sand clay mixtures, Kinetic models, Engineering, Environmental sciences, Solid Earth sciences

## Abstract

This study investigates the hydrogeological and sorption characteristics of clay-sand mixtures as barrier layers in nuclear waste disposal facilities. The primary objective is to evaluate geotechnical properties and the immobilization behavior of strontium (Sr²⁺) and cobalt (Co²⁺) on clay and sand layers under simulated contaminant release scenarios from disposal packages. Saturated hydraulic conductivity (Ks) values for brown clay and brown sand were 3.78 × 10⁻⁸ and 1.02 × 10⁻^3^ cm/s, respectively demonstrating strong resistance to water flow and contaminant migration. Simulated acid water flow significantly increased Ks in brown clay but not in white clay, attributed to kaolinite’s chemical inertness (aluminum silicate-rich) in the latter versus illite, interlayer cations, calcite, and weathering in the former. Batch adsorption experiments and kinetics at pH 6 revealed superior Sr²⁺ and Co²⁺ uptake in brown clay compared to white clay and brown sand. Experimental and modeled adsorption capacities aligned best with the pseudo-second-order kinetic model, outperforming the pseudo-first-order model across all samples.

## Introduction

Radioactive waste is generated through the operations of nuclear power plants, research reactors, and the intricate processes of the nuclear fuel cycle, as well as from a spectrum of activities across industry, research, and medicine that utilize radioactive materials as ^60^Co, ^90^Sr and ^137^Cs and others^1^. Low and intermediate level radioactive waste (LILW), originating from diverse sources, endangers human health through radiation exposure and contamination^1–4^. This approach offers a cost-effective and practical solution for the containment and isolation of radioactive waste, safeguarding the environment and human health from potential hazards associated with this materials^5–8^. The multi-barrier system includes the engineered barriers such as top barrier, waste container, waste form, backfill material, bottom barrier and the natural barrier in sequence^9–14^. The permeability coefficient of the barrier material in the disposal waste is from 10^− 10^ to 10^− 7^ m/s^15,16^ and the effect of the acid infiltration^17,18^.

Radioactive waste contains nuclides like^60^ Co, a highly toxic metal with a γ-emitting product and a half-life of 5.3 years, which can cause diseases such as aplastic anemia and leukemia if ingested^19–21^. ^90^Sr is similar to K and have a β-emitter with a half-life of 28.8 years, are by-products of uranium and plutonium fission. They can enter the environment through nuclear tests, power plant accidents, and waste leaks. Soil contamination by these isotopes has been studied extensively^22–26^. ^60^Co is critically important for waste disposal and remediation^27^. Sorption of Co(II) on soils with varying properties has been widely researched^28–30^, as it affects radionuclide migration in backfill materials. The mobility of cobalt and strontium ions poses groundwater contamination risks^31^, with higher sorption observed in clays containing more clay minerals. Adsorption/ion exchange is a promising, low-cost, efficient method for removing cobalt and strontium due to its selectivity and ease of use^32,33^.

In the study region, the subsurface layers include Quaternary sediments made up of alluvium, sand sheets, Nile silt, and Nile mud. Miocene formations appear in the northeastern and southern sectors, specifically the Hommath Formation, which consists of sandy limestone, sand, sandstone, and clay. Hydrogeological investigations in this area were conducted by DRC (1993), Al-Gamal and El-Messiry (1998), EGSMA (1999), and El-Sayed et al. (2009)^34–37^. The Quaternary aquifer comprises sand and gravel layers interspersed with clay lenses, overlying the Oligocene basalt unconformably in the western part. The Miocene aquifer includes sand, gravel, clay, sandstone, calcareous sandstone, and limestone. Groundwater levels are around 16.0 m (MSL), with the potentiometric surface in the Miocene aquifer beneath the study area rising at 0.206 m/year. Overall, groundwater moves from the Miocene aquifer to the Quaternary one, discharging as baseflow into the Ismailia Canal^38^. Geological and hydrogeological characteristics play a key role in assessing and designing active synthesis barrier using clay and sand layers.

The synthesis barrier typically consists of impermeable materials such as compacted clay^39–41^, geomembranes, or synthetic liners^42–47^. These materials are chosen for their low permeability and high resistance in minimizing of water flow, effectively reducing the potential for leachate formation and the transport of contaminants into the surrounding environment biosphere^48–50^. Hydrogeological parameters such as hydraulic conductivity, porosity, bulk density and pore water velocity are important studies on the probability in a proposed disposal site for the near surface waste disposal Facility^51–53^. The hydraulic conductivity of the barrier layer influences the rate at which water infiltrates through the barrier, impacting the overall performance and longevity of the disposal facility. The Hydrus-1D software is used in the measuring of the unsaturated hydraulic conductivity K(θ) by using the saturated hydraulic conductivity (Ks) and water contents (θ) according to the van Genuchten model^54–56^. The aim of this study is to evaluate clay and various types of sand as potential materials for the barrier layer in near surface waste disposal, assessing their effectiveness involving the mineral components, geotechnical properties to assess waste isolation and environmental protection with studied the behavior of Sr and Co ions with different clay and sand.

## Experimental and methods

### Chemicals and reagents

The chemicals and reagents used in this work were of analytical grade purity and Chloride salts of cobalt and strontium were purchased from Sigma-Aldrich (USA) and Hydrochloric acid (HCl) and sodium hydroxide (NaOH) are from Shanghai Longjin Metal Materials Co., Ltd.). The composition of the simulated acid water is 0.5 M of HCl and alkaline of higher pH 10 (0.5 M NaOH).

### Geochemistry and mineralogy of samples

Five samples were collected from the eastern part of the Delta, Egypt. These included two types of white clay (WC) and brown clay (BC), and three types of sand: white sand (WS), yellow sand (YS), and brown sand (BS). Major and minor oxides were determined using X-ray fluorescence (XRF-1800 SHIMADZU). The XRF instrument is equipped with a rhodium tube and a 2.5 kW generator.

The mineralogy of the samples was analyzed by X-ray diffraction (Philips, XRD-PW1710). This XRD instrument uses Cu radiation (λ = 1.542 Å) with a monochromator, referencing powder diffraction files (PDF-2) as standards, operating at 45 kV, 35 mA, and a scanning speed of 0.03°/s. The morphology of the clay soil samples was examined using a scanning electron microscope (Jeol JSM-6510 A model, Japan). Total surface area of the studied sediments was determined using the BET-equation by means of Nova BET instrument (Quantachrome Corporation, USA), in which N2 adsorption/desorption isotherms were constructed.

The particle size distribution analysis was conducted using the dry sieve analysis method with high precision. Accurate sieve analysis was performed using a F. Kurt Retsch GmbH & Co. KG (Germany) sieve set, following ASTM standards, with sieve diameters of 2.36 mm, 1.70 mm, and 850, 600, 500, 425, 250, 106, and 63 μm. Particle size distribution for the two soils (clay) with particles less than 63 micrometers was determined using the standard hydrometer tests according to ASTM D422-63 (2007) and ASTM D7928-17 (2021)^57,58^. Organic matter (OM) content was measured by loss-on-ignition, where samples were heated to 450°C^59^. Organic matter and carbonate materials were removed using 30% hydrogen peroxide (H₂O₂) and 10% diluted hydrochloric acid (HCl), respectively, following the methods of Ingram (1971)^60^ and Lewis and McConchie (1994)^61^.

### Geotechnical properties of samples

Bulk density, porosity, specific gravity and saturated hydraulic conductivity are the important Geotechnical properties in this study. The bulk volume of the soil samples was determined by measuring the volume of the sampler used to extract the undisturbed soil. Bulk density (*ρ*_*s*_) is expressed in grams of oven-dry soil per cubic centimeter and calculated as *ρ*_*s*_ *= V*_*s*_*/m*, where m is the mass of oven-dry soil (g) and *V*_*s*_ is the bulk volume of soil (cm³)^62,63^. Porosity (*η*) represents the relative amount of pore space in the soil and is defined as the ratio of the volume of voids to the total bulk volume: *η* = *V*_*s*_/*V*_*p*_, where *V*_*p*_ is the volume of void space (cm³). To find *V*_*p*_, the soil sample was saturated with water and weighed. After saturation, the sample was oven-dried for 24 h, and its dry weight was measured. The difference in weight corresponds to the water volume filling the pores.

Specific gravity (*G*_*s*_) of the soil sample was determined using a pycnometer following ASTM D854-14^64^. The pycnometer was cleaned and dried to ensure accuracy. The mass of the dry pycnometer was recorded, then the soil sample was added, and the combined mass measured. The pycnometer was filled with water, avoiding air bubbles, and its total mass recorded. Using these recorded masses, the specific gravity of the soil was calculated according to the formula in Eq. 1:

1 $$G_{s} = \frac{{W_{o} }}{{W_{o} + (W_{a} - W_{b} )}}$$

Where: *G*_*s*_ is the specific gravity; *W*_*o*_ is the weight of sample; *W*_*a*_ is the weight of pynometer with water; *W*_*b*_ is the weight of pynometer with water and sample [64].

2 $$Ks = \frac{{(a.L)}}{{(A.t)}}\ln \left( {\frac{{h_{1} }}{{h_{2} }}} \right)$$

The saturated hydraulic conductivity (Ks) (cm/s) of a soil samples were calculated using Darcy’s law. The constant and falling head permeability test was used for measuring the hydraulic conductivity of fine-grained soils like clay ASTM D5084-16^65^ and Method for Permeability of Granular Soils according to D2434-68^66^. After selecting initial and final heads (h_1_ and h_2_) are according to Laboratory determination of permeability ASTM D2434. After saturation of the soil specimen, four readings were taken to determine the Ks according to Eqs. 2, 3 respectively.

3 $$Ks = \frac{{(V.L)}}{{(A.t.H)}}$$

Where: *V* is the volume collected; *L* is the length of the soil sample (cm); *H* is the constant head; *A* is the cross sectional area of the soil sample (cm^2^); *t* is the time passed (s); *a* is the cross-sectional area of the standpipe (cm2); *h*_*1*_ is initial head (cm), *h*_*2*_ is final head (cm).

**Fig. 1:**
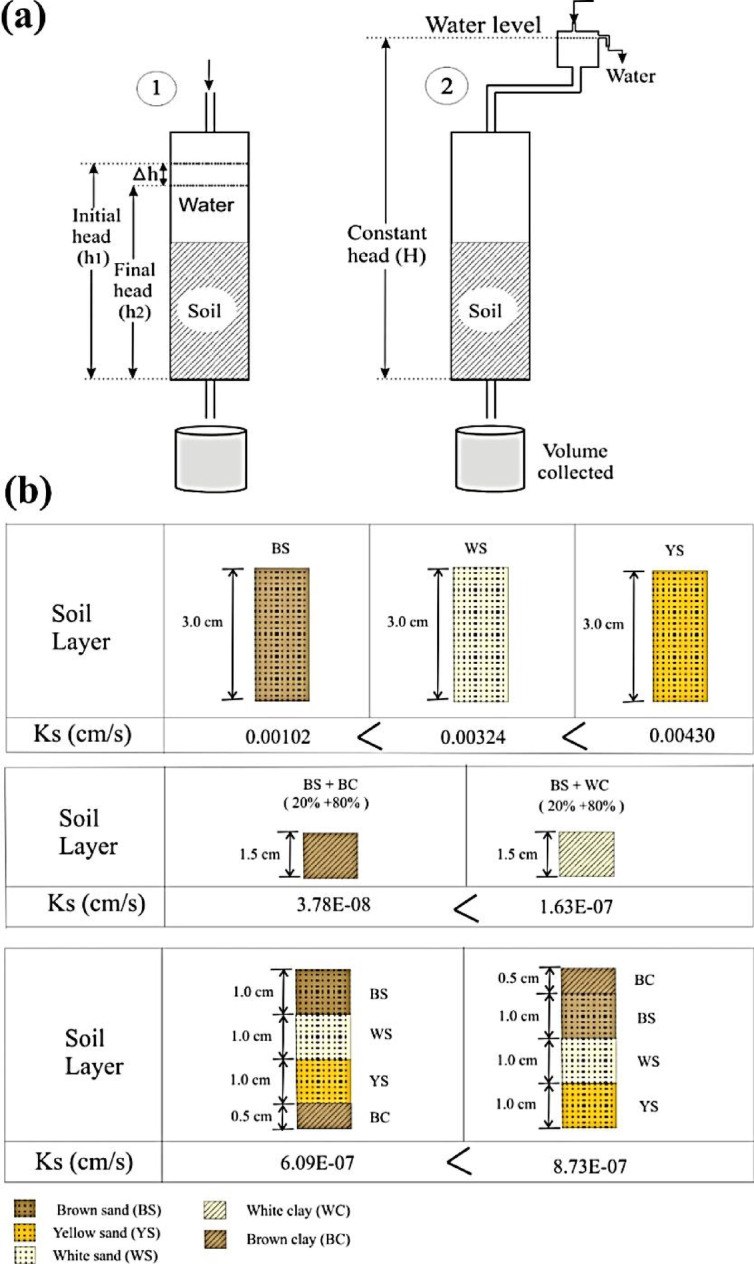
(**a**) Schematic diagram of the experimental hydraulic conductivity, (1) falling head, (2) constant head; (**b**) schematic of stratified soil layers (sand, clay and mixture).

Figure 1(a, b) was illustrated the schematic diagram of the experimental falling and constant head hydraulic conductivity with different stratified layers (sand, clay and mixture). The procedures the hydraulic conductivity of different types of clay and sand only and mixtures determine by placing the sample in a 30 cm length glass column and of an inner diameter of 1.7 cm. The layers of samples are compacted by using a vibrator for 3 min. The saturated hydraulic conductivity (Ks) (cm/s) of sample can be calculated using the following formula:

Hydrus 1D, a simplified version of the Hydrus software, is designed for one-dimensional modeling of water flow and solute transport in variably saturated porous media. The RETC optimization software package^67^ was employed alongside the hydraulic conductivity function at given water content *θ* (Eq. 4), van Genuchten model and is expressed as follows:

4 $$K(\theta ) = Ks.\sqrt {\frac{{(\theta - \theta _{r} )}}{{(\theta _{s} - \theta _{r} )}}} .\left[ {1 - \left( {1 - \left( {\frac{{(\theta - \theta _{r} )}}{{(\theta _{s} - \theta _{r} )}}} \right)^{{(1/n)}} } \right)^{n} } \right]^{2}$$

where, *K*(*θ*) is the unsaturated hydraulic conductivity at a given water content *θ* (cm/s), *Ks* is the saturated hydraulic conductivity, *θ*_*s*_ is the saturated volumetric water content (-), *θ*_*r*_ is the residual volumetric water content (-).

The van Genuchten model is particularly useful in conjunction with Richards’ equation (Eq. 5), which governs the flow of water in unsaturated soils. Richards’ equation can be expressed as:

5 $$\frac{{\partial \theta }}{{\partial t}} = \frac{\partial }{{\partial z}}\left[ {K(h)\left( {\frac{{\partial h}}{{\partial z}} + 1} \right)} \right]$$

Where *θ* is volumetric soil water content (cm^3^/cm^3^); *K(h)* is the hydraulic conductivity, which can be derived from the van Genuchten parameters, *h* is pressure head (cm), *z* is gravitational the head [cm], *t* is time (s). This equation characterizes the relationship between soil moisture content and soil suction, allowing for the prediction of water movement in unsaturated soils was the commonly-applied van Genuchten–Mualem function^68,69^.

### Adsorption experiments

Batch experiments were carried out to investigate the sorption behavior of Sr^2+^ and Co^2+^ onto the three samples of brown clay (BC) and White clay (WC) in particle size less than 63 μm and brown sand (BS) in size less than 500 μm by using sieves (Retsch GmbH& Co. KG 42781 Haan, Germany). The pH on the sorption processes was varied from 2.0 to 6.0 ± 0.1 by contacting 0.1 g each only sample with 10 mL aqueous solution of Sr^2+^ and Co^2+^ (100 mg/l) in 25 mL. The kinetic experimental bottles were placed on the mechanical shaker for overnight at room temperature (25 °C), after which samples were centrifuged for 20 min and determined the residual concentration of strontium and cobalt ions in the supernatant were measured by using atomic absorption spectrophotometer (AAS; Buck Scientific, VGP-210). Kinetic sorption experiments were performed at the optimum sorption in pH 6 because in the higher pH, net negative charge can weaken outer-sphere electrostatic attraction and start to form hydroxides precipitates (e.g., Sr(OH)₂,, Co(OH)₂,) instead of staying as free dissolved ions The amount of metal ion sorbed into natural materials at any time, *q*_*t*_ (mg/g), calculated from the expressions:

6 $$\mathop q\nolimits_{t} = (\mathop C\nolimits_{0} - \mathop C\nolimits_{t} )\left( {\frac{V}{m}} \right)$$

Where, *C*_*o*_ and *C*_*e*_ are the initial and equilibrium concentration of metal ion in solution (mg/l), *V* is the solution volume (*l*) and m is the weight (g) of the sorbent.

## Results

### Geochemistry and mineralogy of samples

**Fig. 2:**
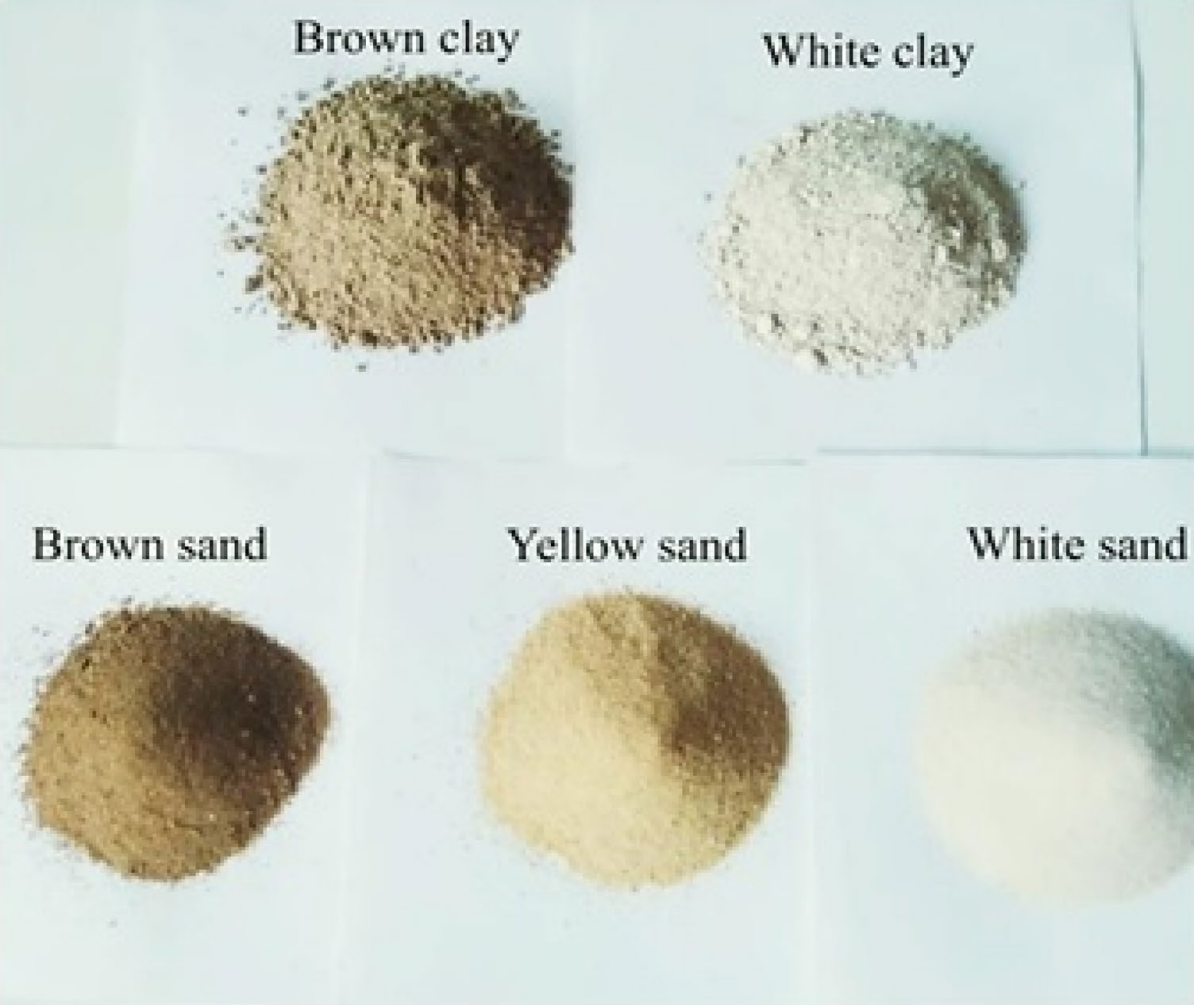
Clay and sand samples used.

The five sediment samples used in this study, including two clay samples and three sand samples, are shown in Fig. 2. The results of the major element analysis for these samples are presented in Table 1. Geochemical analysis revealed that the white sand (WS) contains a high percentage of SiO₂ (97.35%), while yellow sand (YS) and brown sand (BS) have SiO₂ concentrations ranging from 72.04% to 83.22%, respectively. The Al₂O₃ content ranges from 0.53% to 4.57% in the sand samples, but is higher in the clay samples, ranging between 13.7% and 32.57%. The Fe₂O₃ percentage is greater in the brown sand and clay samples, with values of 9.37% and 13.64%, respectively. The relative concentration of major oxides in these samples follows this order SiO_2_> Al_2_O_3_> Fe_2_O_3_> CaO > MgO> Na_2_O> K2O > MnO> SO3 > TiO2.

**Table 1:** Chemical compositions of sand and clay samples determined by XRF (wt%).

Weight%	Sand			Clay	
	WS	YS	BS	WC	BC
SiO _2_	97.35	83.22	72.04	44.68	44.81
Al _2_O_3_	0.53	3.44	4.57	32.57	13.70
Fe_2_O_3_	0.06	2.59	9.37	4.78	13.64
CaO	0.15	2.84	4.31	3.86	5.46
Na_2_O	0.28	2.51	1.92	1.47	1.72
MgO	0.42	1.92	2.76	2.93	4.06
P_2_O_5_	0.09	0.13	0.18	0.18	0.13
K _2_ O	0.06	1.41	1.81	1.68	1.83
TiO _2_	0.29	0.24	0.52	0.55	1.80
SO _3_	0.17	0.37	0.26	1.42	1.74
MnO	0.23	0.16	0.11	0.25	0.29
Cl	0.16	0.15	0.05	0.69	1.86
LOI	0.19	0.98	2.04	4.90	8.90
Total	99.99	99.96	99.94	99.96	99.94

**Fig. 3:**
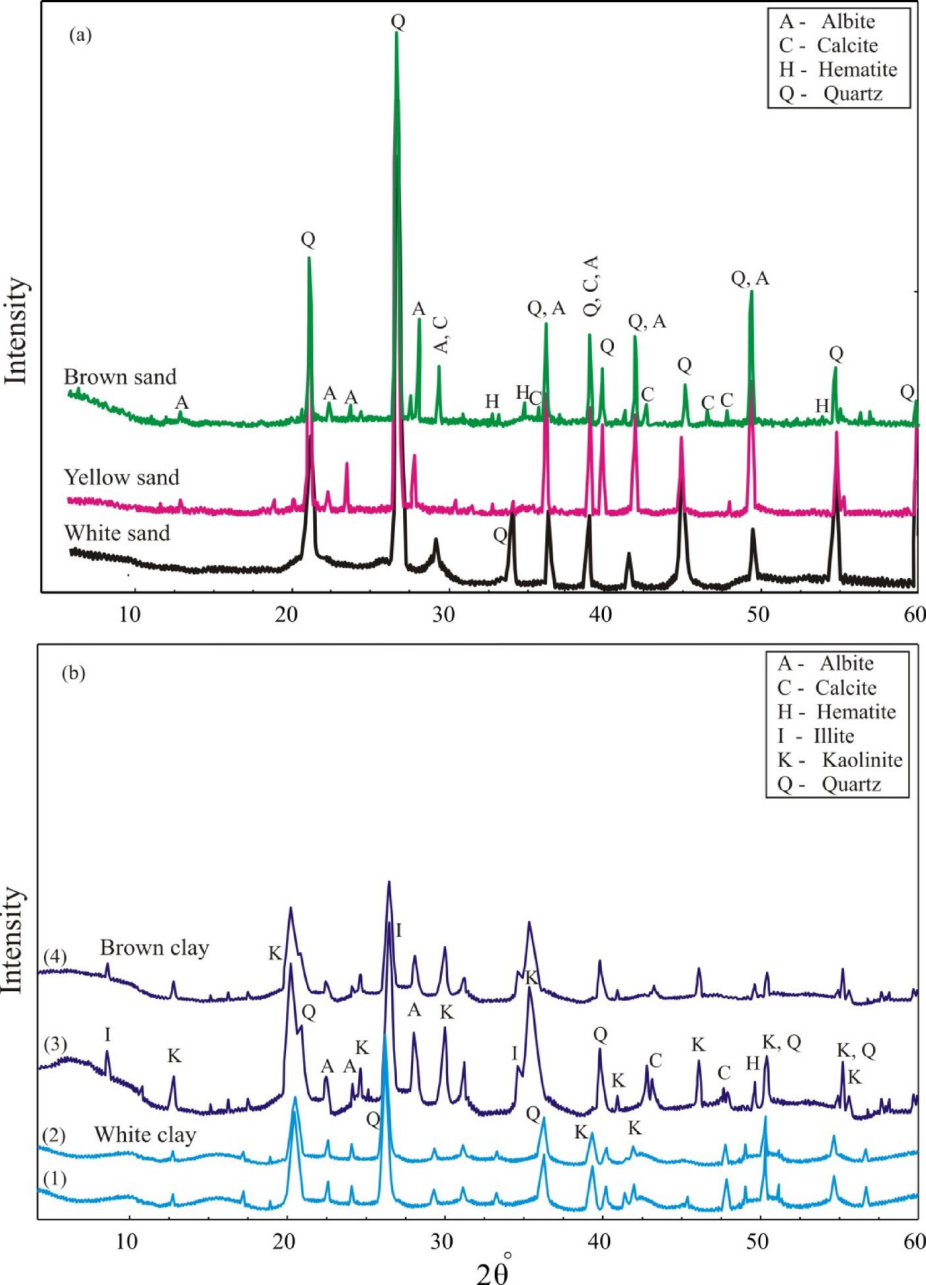
XRD patterns and the mineral composition of the sand and clay samples.

Figure 3 shows the mineralogical composition of the samples as determined by X-ray diffraction analysis. The X-ray diffraction patterns reveal that quartz and albite are the dominant minerals in the white sand (WS) sample. In contrast, hematite and calcite are the main minerals found in the yellow and brown sand samples. The white clay (WC) sample mainly consists of kaolinite, quartz, and albite, while the brown clay (BC) sample contains illite, quartz, hematite, and calcite, as illustrated in Fig. 3(b1,3). Acid treatment progressively reduces the peak intensity observed in clay minerals, primarily due to induced structural disorder that compromises their crystalline structure. Importantly, the clay minerals illite and kaolinite play a key role in regulating water releasing and retention within the barrier layers of disposal facilities. These minerals significantly help prevent water percolation, thereby enhancing the integrity and effectiveness of containment systems^70^. Understanding the mineralogy is crucial for evaluating the physical and chemical stability of the samples in environmental and engineering applications. Treatment with 0.5 M HCl reduces the peak intensity of clay minerals in XRD patterns as shown in Fig. 3(b2,4). These occur as acid leach octahedral cations like Al³, leading to partial amorphization and rearrangement of tetrahedral sheets and disappear on some peaks like calcite. The identification of minerals through X-ray diffraction provides valuable insights into the composition and expected behavior of the materials under different conditions.

**Fig. 4:**
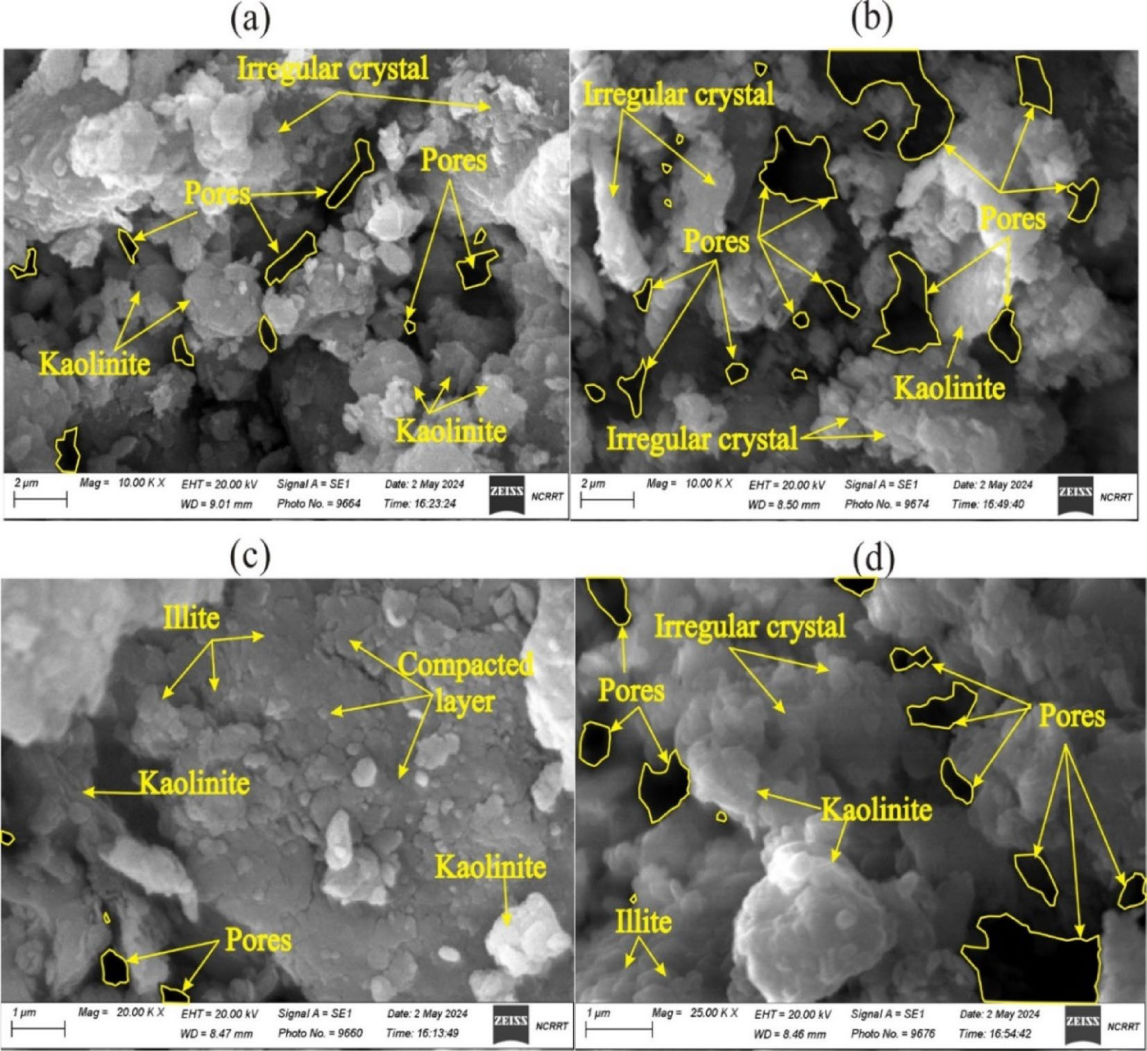
SEM micrograph of (**a, c**) white and brown clay only, (**b, d**) white and brown clay with simulated infiltration of acid water.

The morphology of white and brown clay surfaces shows a combination of compact, regular, and irregular crystal structures, as illustrated in Fig. 4(a, c). The effects of varying pH levels, simulating acidic and alkaline conditions, were studied to assess their impact on these clays. Exposure to simulated acid water, specifically hydrochloric acid (HCl), caused significant damage to the clay minerals. Acid attack primarily disrupted the crystal structures and created cavities, and increasing of permeability.

Acid treatment caused a noticeable reduction in crystallinity across the clay samples, as shown in Fig. 4(b, d). This loss of crystallinity corresponded with an increase in permeability, indicating that acid exposure weakens the structural integrity of the clays and alters their physical properties. These results highlight the sensitivity of clay minerals, especially those rich in calcite, to acidic environments, which can significantly affect their microstructure and hydraulic behavior.

### Geotechnical properties of samples

**Fig. 5:**
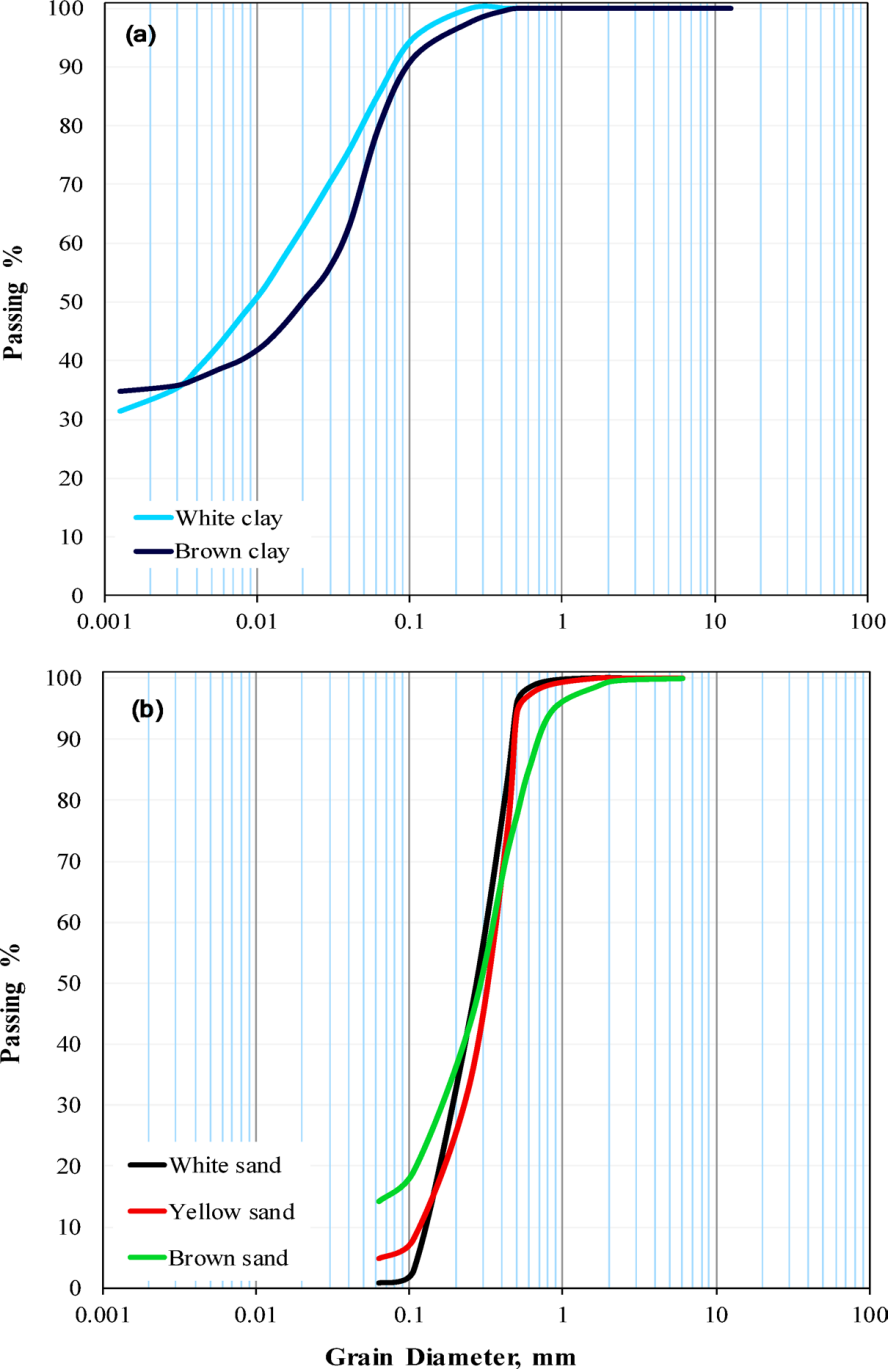
Grain size analyses for (**a**) clay samples, (**b**) sand samples.

The grain size distribution curve are given in Table 2; Fig. 5 and the degradation of the grain size in WS is between fine and medium size while the larger percentage of the medium grain size is in the YS. The BS has the larger percentage of medium to coarse sand with fine grain size (< 63 μm) is 14.12% which effect on the impermeability and infiltration of sand layer and the BC has the larger percentage than WC of clay (less than 2 μm) is 35.14%. The coefficient of uniformity (C_u_) and coefficient of curvature (C_c_), defined as C_u_ = D60/D10 and C_c_ = D30^2^/D10 xD60 were calculated based on the grain size distribution curve. A higher value of C_u_ indicates a broader range of particle sizes, suggesting a well-graded soil. Typically, a coefficient of uniformity (C_u_) values greater than 4 to 6 denotes well graded soil, while the value less than 4 indicates poorly graded or uniformity graded soil.

**Table 2:** Distribution of grain sizes of gravel, sand, silt and clay samples.

Grain size		Sand Wt. %			Clay Wt. %	
		WS	YS	BS	WC	BC
Granule gravel (> 2 mm)		-	-	0.320	-	-
Sand (2–63 μm)	Coarse	4.00	5.60	22.08	-	-
	Medium	50.96	60.16	33.88	-	2.33
	Fine	44.24	29.44	29.60	14.40	17.67
(< 63 μm)	Silt	0.80	4.80	14.12	54.07	44.86
	Clay				31.53	35.14

The soil classification parameters are given in Table 3, which indicates the BS is well graded soil but in YS and WS are poorly graded. Coefficients of curvature (Cc) typically are ranged between 1 and 3, the soil classified as well graded as found in the YS and BS.

**Table 3:** Soil classification parameters of sand used in this study.

Samples	Particle size distribution [ASTM D422-63]				
	D_10_	D_30_	D_60_	C_u_	C_c_
White sand	0.124	0.184	0.31	2.50	0.88
Yellow sand	0.114	0.217	0.345	3.03	1.20
Brown sand	0.063	0.155	0.356	5.65	1.07

Table 4 presents the density values of the sand and clay samples, showing that sand densities range from 1.62 to 1.66 g/cm³, while clay densities are lower, ranging from 1.44 to 1.48 g/cm³. It is observed that hydraulic conductivity tends to increase with sample density. Table also summarizes specific gravity values, indicating that clay samples have higher specific gravity, ranging from 2.63 to 2.73, compared to the sand samples. Porosity varies between 0.31 and 0.45, significantly influencing hydraulic conductivity in sand samples. However, in sand and clay samples, hydraulic conductivity appears largely depend on the total pore volume. The density and apparent porosity are not limiting the direct relationship between porosity and hydraulic conductivity on clay and sand.

**Table 4:** The density, specific gravity (G_s_), porosity and hydraulic conductivity (Ks) of sand and clay samples.

Parameters	Sand			Clay	
	WS	YS	BS	WC	BC
Density (g/cm^3^)	1.65	1.62	1.66	1.44	1.48
specific gravity (G_s_)	2.68	2.63	2.65	2.71	2.73
Porosity	0.35	0.37	0.32	0.45	0.42
Ks (cm/s)	0.00324	0.0043	0.00102	1.56E-06	6.09E-07

### Sorption investigation

**Fig. 6:**
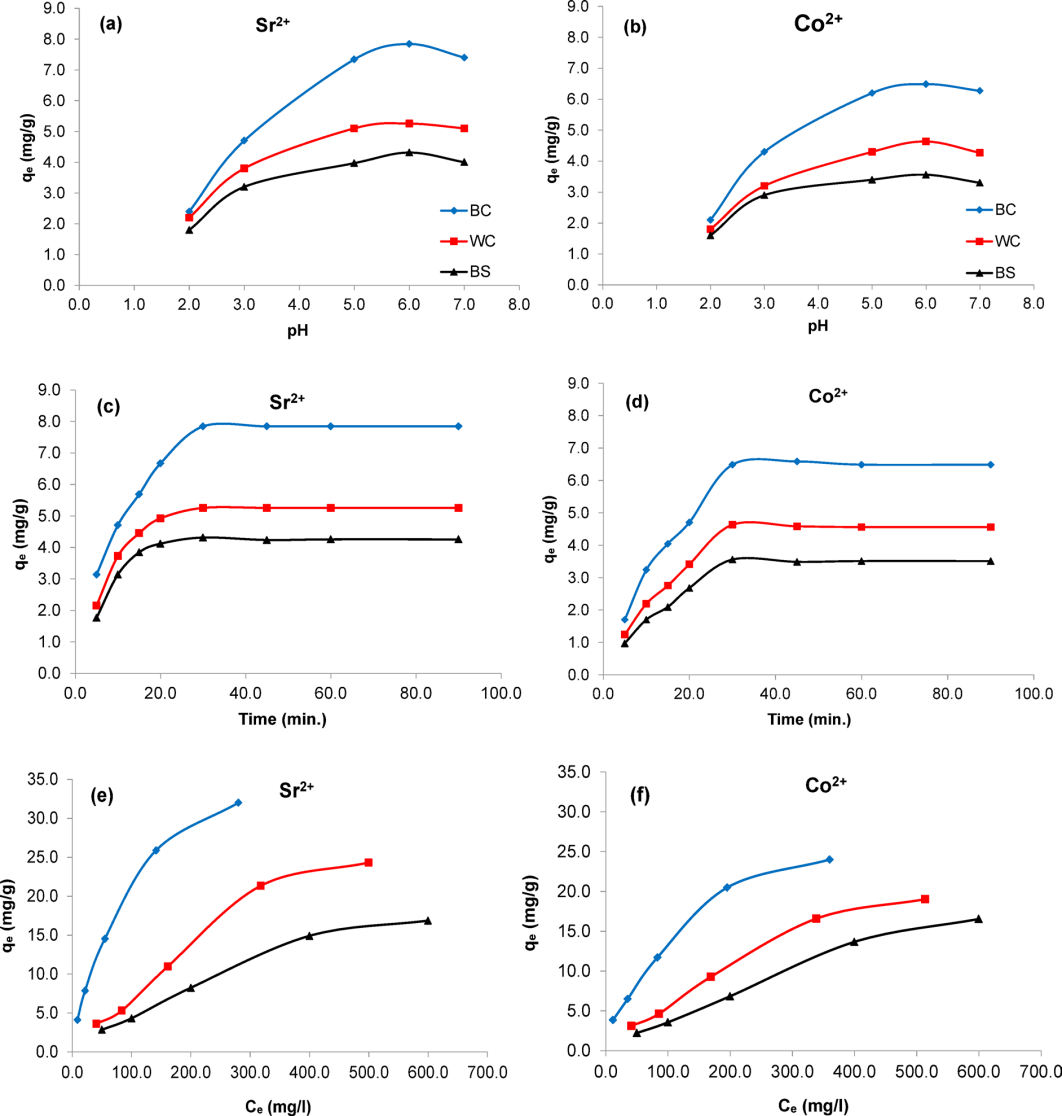
batch studies of Sr^2+^ and Co^2+^ sorption onto BC, WC and BS (**a, b**) Effect of the aqueous solution pH, (**c, d**) Effect contact time and (**e, f**) Isotherm of adsorbate concentration at equilibrium.

The sorption of Sr^2+^ and Co^2+^ from aqueous chloride solution was tested experimentally to investigate the sorption capacities of eight samples from the sediments in the study area as behavior of the radionuclides released into barrier layer (geosphere). The objective of this part of the study to investigated the kinetic models and their parameters for the sorption of Sr^2+^ and Co^2+^ onto BC, WC and BS without using the white sand (WS) because it was as the drainage without any sorption process. In Fig. 6(a, b), It was observed that an increasing of amount sorbed of Sr^2+^ and Co^2+^ by increasing of pH 6 and decrease at pH7. The experimental data showed that the amount of the metal ions sorbed increases gradually with time (5–90 min range), and then reach an equilibrium value at 30 min. The effect of time at initial metal ion concentration (100 mg/l) with optimum pH 6 ± 0.1 for Sr and Co ions illustrated as shown in Fig. 6(c, d). The sorption in BC sample is the higher than from the other samples of WC and BS, which based on their capacity and low permeability to limit transfer towards the biosphere of any radioactive elements released from the waste substances. Sorption equilibrium is usually described by the relation between the amount of adsorbate (*q*_*e*_) on the adsorbent and the concentration of dissolved adsorbate in the liquid at equilibrium (*C*_*e*_) for ions as was shown in Fig. 6(e, f).

### Kinetic modeling

Sorption kinetics is controlled by different kind of mechanisms like mass transfer, diffusion control, chemical reactions and particle diffusion. The pseudo-first-order^71^ and pseudo-second-order rate^72^ and Elovich models^73^ include all steps of sorption in order to identify the controlling sorption mechanism. In order to clarify the kinetic characteristics of sorption of Sr^2+^ and Co^2+^ sorption onto three samples of BC, WC and BS with the time, an appropriate kinetic model is required. The statistical comparisons of correlation coefficient (*R*^2^), a number of error functions such as root mean square error (RMSE) used to rule the equilibrium model that calculated and sum of squared errors (SSE).

#### Pseudo-first-order kinetic model

 The pseudo-first-order kinetic model describes the sorption rate based on the sorption capacity. The model assumes that the reaction rate is limited by only one process or mechanism on a single class of sorbing sites and that all sites are of the time dependent type. The Lagergreen pseudo-first-order expression as written as:

7 $$\frac{{{\mathrm{d}}q_{t} }}{{{\mathrm{d}}t}} = k_{1} (q_{e} - q_{t} )$$

After the integration, the form of Eq. (8) becomes:

8 $$log\left( {q_{{e}} - q_{{t}} } \right) = logq_{{e}} - \frac{{k_{1} }}{{2.303}}t$$

**Fig. 7:**
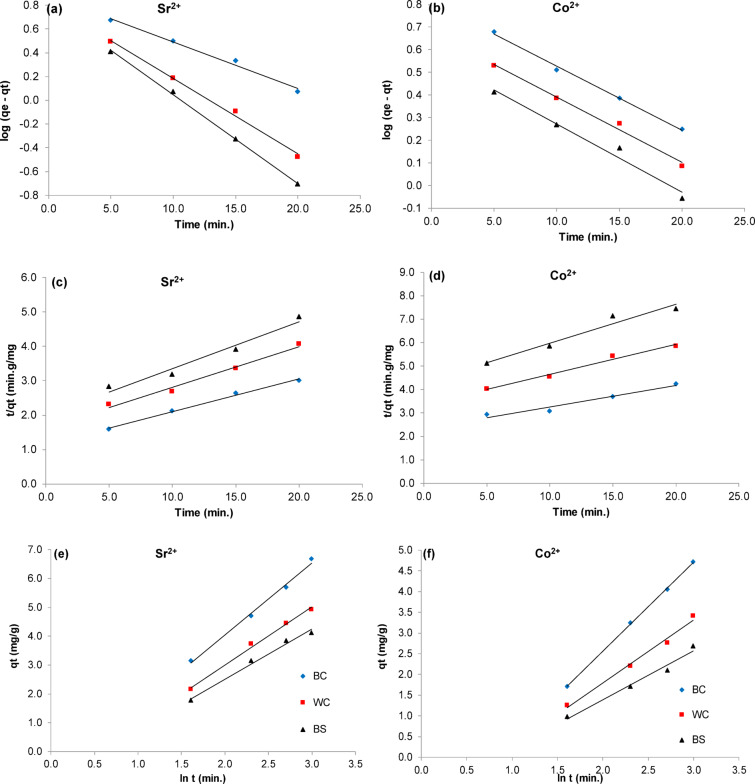
Kinetic studies of Sr^2+^ and Co^2+^ sorption onto BC, WC and BS (**a, b**) pseudo-first-order, (**c, d**) pseudo-second-order and (**e, f**) Elovich kinetic model at T (25 ± 2.0 °C), pH (6 ± 0.1).

Where, *q*_e_ and *q*_t_ (mg/g) are the amount of metal ion sorbed onto geological materials at equilibrium and at time t, respectively and *k*_1_ is the pseudo-first-order rate constant (min^− 1^). The plotting of log (*q*_e_-*q*_t_) versus time for Sr and Co ions sorption onto the samples are shown in Fig. 7(a, b), which used in determine the first order rate constant (*k*_*1*_) and the theoretical equilibrium sorption capacities (*q*_*e*_), from the slopes and intercept respectively. Table 5 shows that the results of kinetic studies of Sr^2+^ and Co^2+^ sorption onto the BC, WC and BS soil samples.

#### Pseudo-second-order kinetic model

 A pseudo-second-order rate model is also used to describe the kinetics of the sorption of ions sorbed onto adsorbent materials. This model is expressed as:

9 $$\frac{t}{{q_{t} }} = \frac{1}{{k_{2} q_{e} ^{2} }} + \frac{1}{{q_{e} }}t$$

Where *k*_2_ is the rate constant of pseudo-second-order equation (kg/mg min). The kinetic plots of *t/q*_t_ versus *t* for Sr^2+^ and Co^2+^ sorption onto BC, WC and BS soil were shown in Figs. 7(c, d). The linear relationship and the values of *R*^2^ to 0.99 to 0.95 and the lower values *RMSE* observed that the *q*_e_ (calculated) values for Sr^2+^ and Co^2+^ are in agreement with *q*_e_ (experimental) explain that the process of sorption of ions follows the pseudo-second-order kinetic model. The products *k*_2_*q*_e_^2^ is the initial sorption rate which presented as *h = k*_2_*q*_e_^2^. The kinetic parameters of this model were calculated from the slopes and intercept in Table 5. It is possible to suggest that the sorption of Sr^2+^ and Co^2+^ followed the pseudo-second-order kinetic model for three samples that the overall rate constant of each sorption process appears to be controlled by chemical sorption process^72^. The calculated and experimental adsorption capacity for pseudo-second-order, and pseudo-first-order models, were indicated that it better describes the adsorption kinetics of Sr^2+^ and Co^2+^ onto clay and sand samples.

#### Elovich kinetic model

Elovich kinetic model was used to describe the second-order kinetics if the adsorbent surfaces are energetically heterogeneous^73^. Elovich equation has been widely applied in adsorption kinetics, which Elovich equation is generally applicable for chemical adsorption onto a highly heterogeneous surface^74^, the linear form of Elovich equation is expressed by Eq. (10).

10 $$q_{t} = \frac{{1_{{}} }}{{\beta _{{}} }}\ln (\alpha \beta )_{{}} + \frac{{1_{{}} }}{{\beta _{{}} }}\ln t_{{}}$$

Where α and β are the Elovich constants. α (mg/g min) represents the rate of chemisorption at zero coverage and β (g/mg) is related to the extent of surface coverage and activation energy for chemisorption were determined from the slope and intercept of the plot of qt versus ln (t) as presented in Fig. (7e, f), the plot of q_t_ versus ln (t) was found to be linear with a good determination coefficient, R^2^. The *β* value was higher in brown sand (BS) but α value was higher in brown clay (BC) for Sr and Co ions as shown in Table 5.

**Table 5:** Pseudo**-**first**-**order, pseudo**-**second**-**order, and Elovich kinetic models parameters for Sr^2+^ and Co^2+^ sorption onto BC, WC and BS at T (25± 2.0^o^C), pH (6± 0.1).

**Model**	**parameters**	Sr^2+^			Co^2+^		
		**BC**	**WC**	**BS**	**BC**	**WC**	**BS**
Pseudo-first-order	_e_, _exp_ (mg/g)	**7.84**	**5.25**	**4.31**	**6.49**	**4.63**	**3.56**
	q_e_, _cal_ (mg/g)	7.690	6.640	6.240	6.460	4.800	3.758
	*k*_1_, (min^-1^)	0.091	0.147	0.172	0.065	0.067	0.070
	R^2^	0.987	0.996	0.998	0.996	0.990	0.976
	*SSE*	0.005	0.005	0.008	0.0005	0.001	0.001
	*RMSE*	0.0339 0.032	0.0346	0.0459	0.0110	0.0157	0.0179
Pseudo-second-order	*q*_e_, _cal_ (mg/g)	10.571	8.439	7.369	10.905	7.813	6.035
	k_2_, (g/mg .min)	0.008	0.009	0.009	0.004	0.005	0.006
	*h*, (mg/g .min)	0.865	0.614	0.500	0.426	0.297	0.231
	R^2^	0.993	0.982	0.962	0.952	0.982	0.954
	*SSE*	0.002	0.012	0.028	0.067	0.079	0.117
	*RMSE*	0.023	0.055	0.084	0.129	0.141	0.171
Elovich kinetic	*β* (g/mg)	0.401	0.499	0.575	0.465	0.658	0.849
	*α* (mg/g min)	1.706	1.213	0.999	0.956	0.667	0.519
	R^2^	0.993	0.994	0.987	0.999	0.988	0.978
	*SSE*	0.064	0.051	0.042	0.026	0.016	0.014
	*RMSE*	0.126	0.113	0.103	0.081	0.063	0.059

### The Hydraulic conductivity of samples

**Fig. 8:**
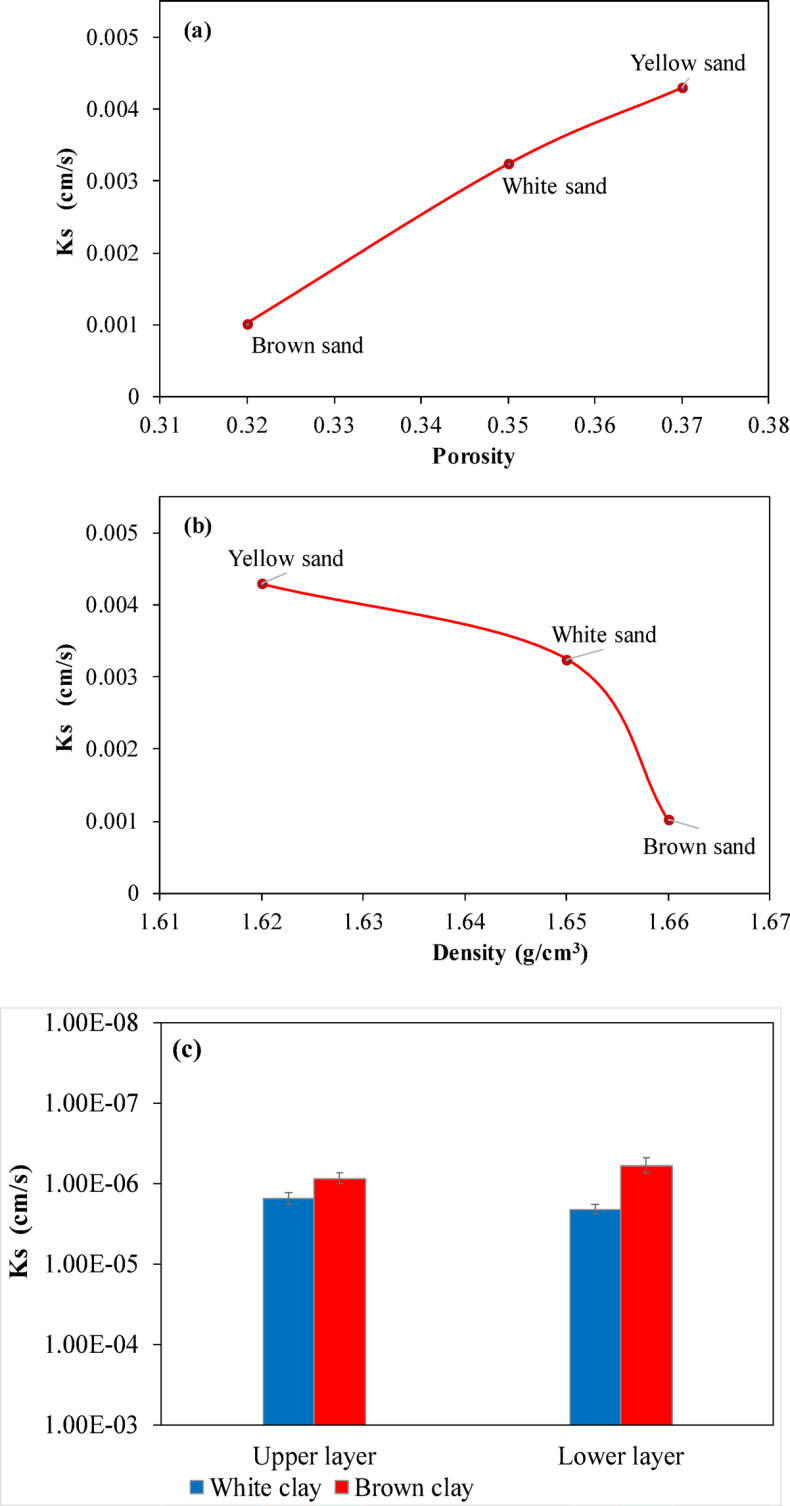
The hydraulic conductivity of WS, YS and BS individually layer with [(**a**) porosity and (**b**) density], (**c**) Three stratified layers of WS, YS and BS with upper and lower WC and BC layer.

The porosity effect on the saturated hydraulic conductivity (Ks) of the particle of sand size as according to the Fig. 8, and the brown sand (BS) have the less than porosity of white and yellow sand samples. The fine gain size of particles (less than 75 μm) was effect on the retention and impermeability of the sand samples (1.0 cm for each layer) as BS > WS > YS (0.00102, 0.00324, 0.00430 cm/s), respectively. Figure 8b indicated that the hydraulic conductivity in the stratified of these (BS, YS and WS) sand samples with depth 3.0 cm (1.0 cm for each layer) with white or brown clay samples by depth 0.5 cm (in upper and lower of sand layers). The values of Ks in the sand layers are suitable as drainage layers according to^75^. The upper layer of white clay had lower permeability than its lower layer, while the lower layer of brown clay had lower permeability than its upper layer.

**Fig. 9:**
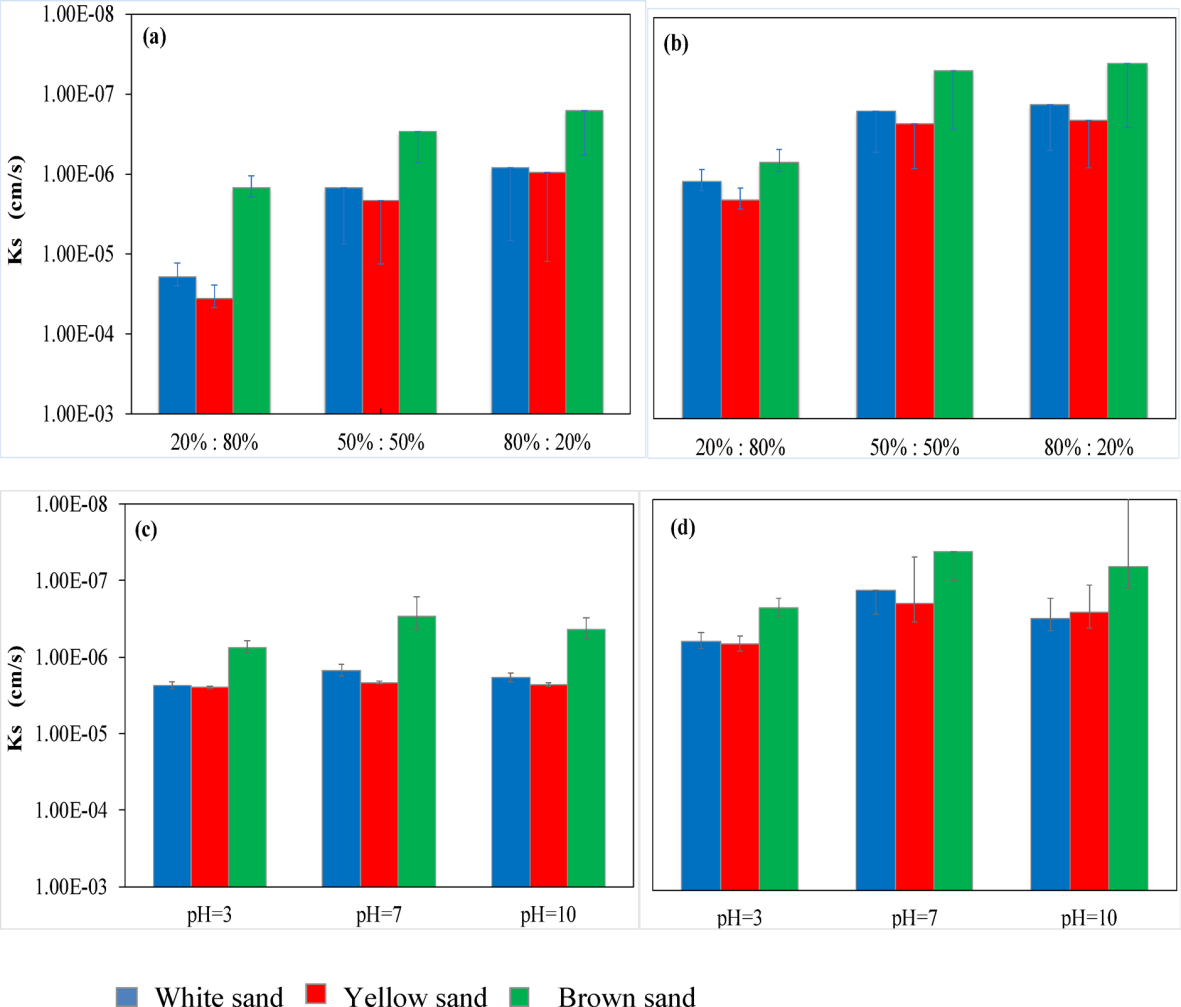
hydraulic conductivity of (**a**) White clay, (**b**) Brown clay individually mixtured with WS, YS and BS] at pH = 7 and different percetage, (**c**) White clay, (**d**) Brown clay individually mixtured with WS, YS and BS at different of pH (3, 7, 10).

In the Table 6, hydraulic conductivity (Ks) of white clay (WC) was mixed with brown sand (BS) as stratified layer 1.5 cm is 1.63 × 10^− 7^ cm/s. The hydraulic conductivity of brown clay (BC) was 3.78 × 10^− 8^ cm/s in mixed with BS is represent the good of hydraulic conductivity about mixed layers as Fig. 9(a, b). the Ks is suitable to barrier layer in the near-surface disposal of low and intermediate level radioactive wastes according^75^ and German regulations take it as 1 × 10^− 6^ and 1 × 10^− 7^ cm/s, respectively. The effect of simulated acid water on increasing of the hydraulic conductivity of clay mixture with different sand samples because the acid solution caused the increasing of the total pore volume and a voids between the particles on layers with dissolution in formation of cracks^76–78^. In Fig. 9(c, d) and Table 6 shows that hydraulic conductivity of BS and BC at the same percent increases at pH 3 and pH 10. Acid treatment elevates the specific surface area and total pore volume of clay that increase of clay permeability as illustrated in Table 7.

**Table 6:** Hydraulic conductivity of WC and BC with WS, YS and BS at different percentage at (pH = 7), and different of pH in the same percentage of clay and sand (50: 50%).

**Clay: sand**		**hydraulic conductivity (Ks), ** **cm/s**					
	**pH**	**WC**			**BC**		
		**WS**	**YS**	**BS**	**WS**	**YS**	**BS**
20: 80%	7.0	1.93E-05	3.60E-05	1.49E-06	1.12E-06	1.89E-06	6.41E-07
80: 20%	7.0	8.37E-07	9.64E-07	1.63E-07	1.23E-07	1.93E-07	3.78E-08
50: 50%	7.0	1.49E-06	2.13E-06	2.95E-07	1.47E-07	2.12E-07	4.64E-08
50: 50%	3.0	2.33E-06	2.47E-06	7.45E-07	6.54E-07	7.15E-07	2.45E-07
50: 50%	10.0	1.84E-06	2.28E-06	4.38E-07	3.32E-07	2.81E-07	7.13E-08

**Table 7:** Specific surface area and total pore volume of clay and effect of the acid treatment.

Sample	Acid treatment	Total Pore Volume (cm^3^/g)	BET Surface Area. S_BET_ (m^2^/g)
**White clay**	**before**	0.14	40.3
	**After**	0.18	54.8
**Brown clay**	**before**	0.11	31.5
	**After**	0.21	58.3

### Numerical modeling of unsaturated hydraulic conductivity

Numerical modeling of unsaturated hydraulic conductivity K(θ) and water retention θ(h) in unsaturated zone were simulated using HYDRUS-1D. The unsaturated hydraulic conductivity was predicted using the experimental data of Ks using the Van Genuchten (1980) models^69^. The experimental data of density, percentage of coarse and fine grain size and Ks were used in RETC program to estimate unknown parameters related to retention and unsaturated conductivity functions, as outlined in Table 8.

**Fig. 10:**
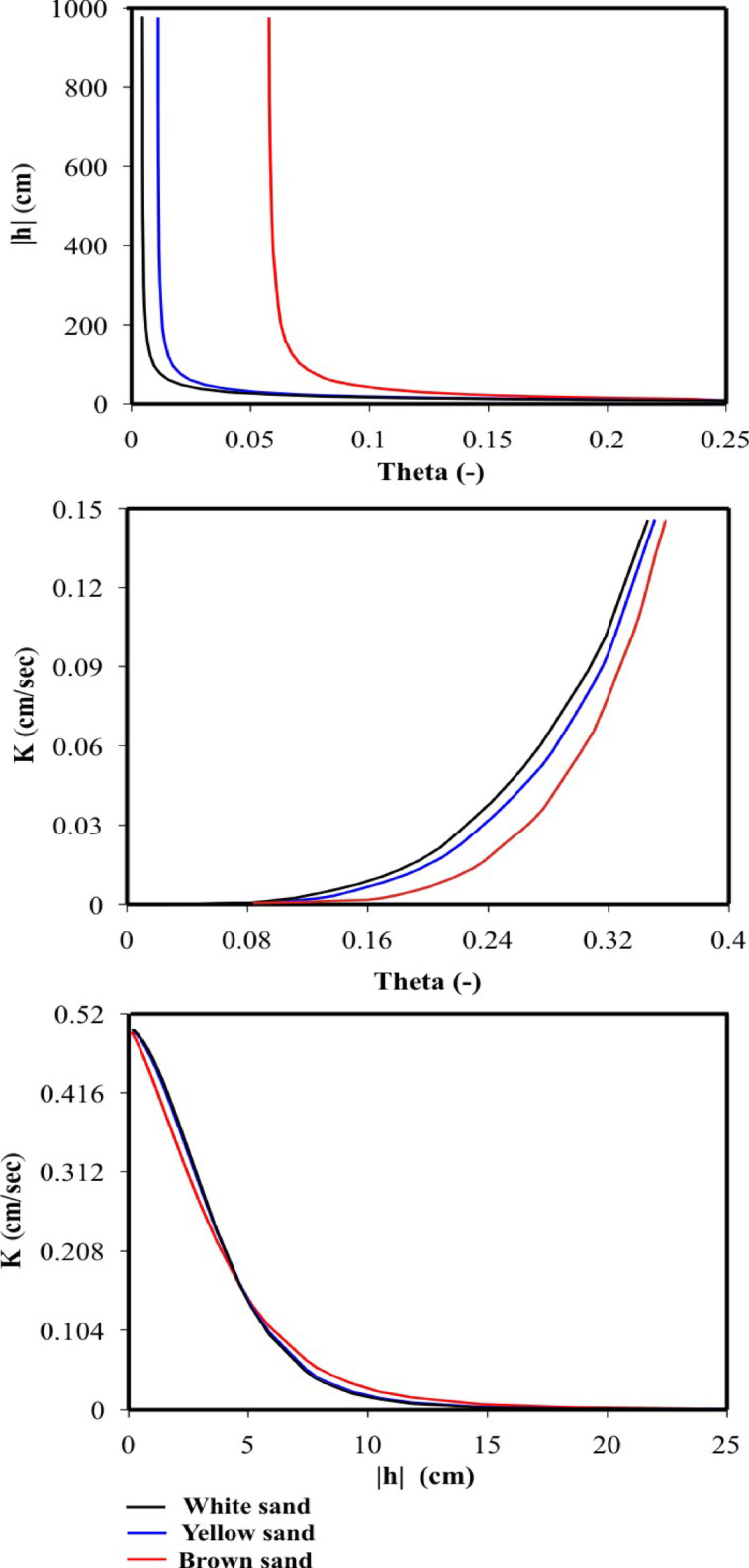
The estimation of the water content, hydraulic conductivity (K) and the pressure head in three sand samples of WS, YS and BS.

**Fig. 11:**
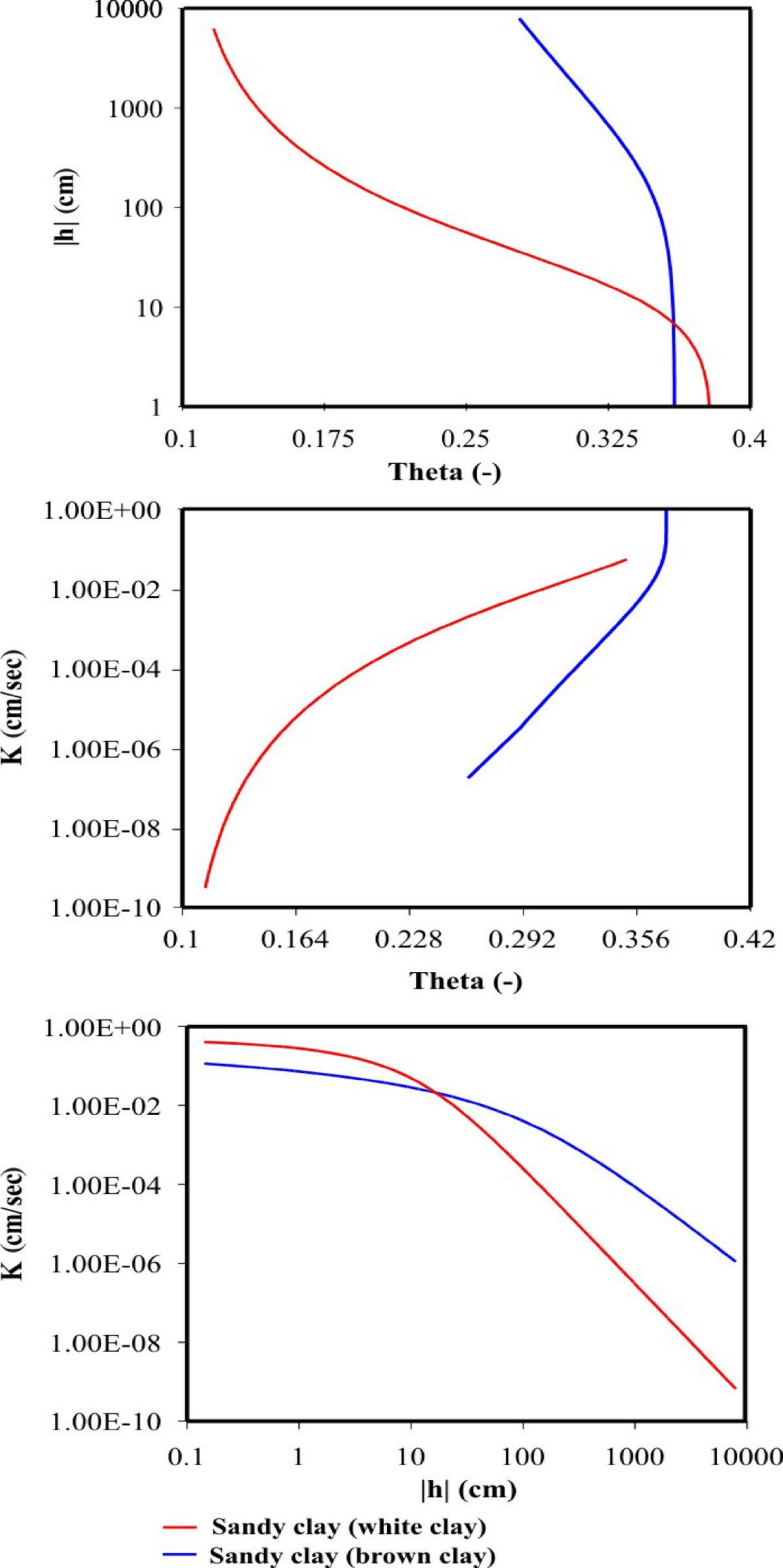
The estimation of the water content, hydraulic conductivity (K) and the pressure head in different samples of BS with WC and BC mixtures in percentage (20% for sand, 80% for clay).

In the Figs. 10 and 11 were illustrated the volumetric soil water contents and their corresponding pressure heads (h) and the effect of the water content on the saturated hydraulic conductivity (K_s_) for three types of sand samples (WS, YS and BS) individually and mixture clay samples (WC and BC). The increasing in the water content (θ) led to decrease in the pressure head and the values of residual water contents (θ_s_) in sand samples is higher with increasing the fine grain size percentage as BS > YS > WS. Residual water contents (θ_s_) in BC with sand mixture is higher than in the mixture of WC with sand. Additionally, while hydraulic conductivity increases with increasing in the water content (θ) of the soil and it exhibits an inverse relationship with pressure head. This behavior is primarily influenced by the distribution of particle sizes within the soil matrix.

**Table 8:** Soil hydraulic parameters used for numerical simulations and parameters for sand and clay samples.

Soil	θ_*r*_ (-)	θ_s_ (-)	α (cm^− 1^)	*N* (-)	Ks (cm/s)
WS	0.0045	0.43	0.145	2.68	0.00324
YS	0.011	0.43	0.141	2.60	0.0043
BS	0.057	0.41	0.124	2.28	0.00102
WS + WC (20, 80%)	0.10	0.38	0.059	1.48	2.95E-07
BS + BC (20, 80%)	0.07	0.36	0.005	1.09	4.64E-08

## Conclusion

This study demonstrated that the hydraulic conductivity of soil samples is strongly influenced by grain size distribution, mineral composition, and environmental conditions such as infiltration of acid and alkaline water. Brown clay (BC) as individually layer is lower permeability of 6.09 × 10^˗7^ cm/s, which may cause water to drain and collect in the yellow sand (YS) layer. The mixture of brown clay and brown sand had the lowest Ks value in the study, at 3.78 × 10^˗8^ cm/s. The lowest hydraulic conductivity was found in brown sand (BS) due to its high fine grain content of 14.2%. The behavior of the Sr and Co ions onto BC, WC and BS were more suitable for pseudo-second-order, and pseudo-first-order models in kinetic absorption description. The effect of varying pH levels, white clay (WC) contains kaolin, mainly aluminum silicate, which is resistant to acid due to its inert nature. While, the brown clay (BC) contains illite and calcite, making it more susceptible to acid weathering and swelling when exposed to acidic conditions. Simulated acid water increased the hydraulic conductivity for both brown and white clay with brown sand mixture. Acid treatment elevates on a specific surface area and total pore volume for clay. Consequently, clay permeability rises as well. Hydraulic conductivity was influenced by soil texture, grain and pore size distribution, structure, and moisture content. Future work could modify the clay to enhance its adsorption capacity for Sr and Co ions of the present soil from clay to make the more active of the barrier layer in adsorption of contaminants of Sr and Co ions.

## Data Availability

Yes (The datasets used and/or analysed during the current study available from the corresponding author on reasonable request).
